# Concomitant Boost With Six Fractions of Radiation a Week in Locally Advanced Head and Neck Cancer Patients: A Prospective Study

**DOI:** 10.7759/cureus.67916

**Published:** 2024-08-27

**Authors:** Winsome Kumar, Vikas Yadav, Jaspreet Kaur, Ratika Gupta, Anu Agrawal, Kapil Suri, Akhilesh Mishra, Ankit Dhameliya

**Affiliations:** 1 Department of Radiation Oncology, Vardhaman Mahavir Medical College and Safdarjung Hospital, New Delhi, IND

**Keywords:** six fractions a week, concomitant accelerated chemoradiation, accelerated radiation therapy, head and neck cancer, concomitant boost

## Abstract

Background and objective

Radiation therapy plays a significant role in the radical treatment of locally advanced head and neck cancers. Studies have shown the radiobiological advantage of accelerated chemoradiation over conventional chemoradiation as it reduces the chances of accelerated repopulation and decreases overall treatment time. This study aimed to assess the response and toxicities of accelerated concomitant chemoradiation in locally advanced head and neck cancer patients.

Methods

A total of 51 patients were enrolled and treated with accelerated concomitant chemoradiation, receiving one fraction of radiation per day, six fractions per week, with the sixth fraction as a boost on Saturdays, with weekly concurrent cisplatin at 40 mg/m^2^. Patients were followed up till six months after treatment completion. Radiological investigation was done to assess response according to Response Evaluation Criteria in Solid Tumors (RECIST) 1.128, and acute toxicities were assessed according to Radiation Therapy Oncology Group (RTOG) criteria.

Results

The median follow-up period was six months; 28 patients (62.22%) had a complete response and 17 (37.78%) had a partial response at six months post-completion of the treatment. The maximum acute toxicities developed at the completion of treatment. Grade III and IV mucositis developed in 14 patients (31.11%) and grade III dermatitis developed in one patient (2.22%), without any grade IV dermatitis during the total duration of treatment. The toxicities were manageable, and most of them resolved after three months of treatment completion.

Conclusions

Accelerated concomitant chemoradiation with six fractions of radiation in a week led to a decrease in overall treatment time. Of note, 62.22% of patients had complete remission, with manageable acute mucositis and dermatitis, which resolved in 82% and 67%, respectively within three months of treatment completion. However, further studies involving larger samples and longer follow-ups are needed for this regimen to be established as the standard of care in the future.

## Introduction

Carcinoma of the oral cavity, pharynx, and larynx form a major component of head and neck malignancies in which squamous cell carcinoma (SCC) is the most common histology [1]. Each year, approximately 6,000 patients are diagnosed with head and neck squamous cell carcinoma (HNSCC) worldwide. India has the highest oropharyngeal cancer incidence in the world with more than 1,000,000 cancer patients registered annually [2].

Chemoradiation therapy (CRT) is considered the primary treatment for locally advanced carcinoma of the oropharynx, larynx, hypopharynx, and unresectable oral cavity carcinoma [3]. Locoregional failure is the predominant recurrence pattern and most deaths result from uncontrolled regional disease and local disease recurrence. One of the theories that explain this locoregional failure is accelerated repopulation, which suggests that after an initial cytoreduction of tumor volume, the radio-resistant tumor cells start to multiply at rapid rates, forming monoclonal colonies of radio-resistant cells. Hence, the cure rates for SCC are highly dependent on overall treatment time [4]. Each extra day of treatment requires approximately 0.6 Gy of extra radiation dose to offset potential regeneration, suggesting a doubling time of three and a half to five days [5-7]. Especially for HNSCC, it is now well-documented that if the overall treatment time is prolonged, for each extra day, the local control decreases by 1.4% (range: 0.4 to 2.5%) [8]. Hence, the decrease in overall treatment time can increase the probability of local tumor control.

Several studies have shown an increased control of local disease with accelerated radiation therapy regimens [9-11]. Various accelerated radiation therapy regimens that have been used are accelerated hyperfractionation, CHART (continuous hyperfractionated accelerated radiotherapy), radiation therapy with concomitant boost, and split-course hyperfractionation [2,11-15]. The Danish Head and Neck Cancer Group (DAHANCA) 6 and 7 compared conventional five fractions of radiation per week with six fractions of radiation per week (sixth fractions as a concomitant boost on Saturdays) as a method of acceleration and concluded that six fractions have statistically significant better five-year locoregional control and disease-specific survival than five fractions per week [15].

There is scarce data available in India regarding accelerated schedules. In light of this, we performed an accelerated fractionation schedule delivering six fractions of radiation in a week with a concomitant radiation boost on Saturdays instead of the conventional five fractions per week with the same dose per fraction and same total dose, which led to reduced overall treatment time.

## Materials and methods

This was a single-institution, prospective study of 51 patients of locally advanced head and neck carcinoma conducted for a duration of 18 months, from August 2022 to January 2024, to assess the response and toxicities of accelerated concomitant CRT. The study was approved by the Institutional Ethical Committee. After a detailed clinical history and examination, biopsy-proven locally advanced SCC of head and neck patients with stage III and IV aged 18-70 years having Eastern Cooperative Oncology Group (ECOG) performance status of 0-2 were selected for the study. Patients who received any oncological treatment in the past or had recurrent or metastatic disease or second primary and tumors of the nasopharynx and PNS tumors were excluded. The primary endpoint of the study was to assess the response to the treatment at the first, third, and sixth months post-treatment. The secondary endpoint was to assess the acute mucositis and dermatitis every week during treatment, at the first and third months post-treatment completion.

Treatment

Informed written consent was obtained from each patient before starting the treatment. Investigations done were as follows: ENT evaluation - direct/indirect laryngoscopy; histopathology - biopsy from the primary site; blood investigations - complete blood counts, routine biochemistry investigations [liver function test (LFT), renal function test (RFT), serum electrolyte (SE), random blood sugar (RBS)]; imaging for evaluation - [contrast-enhanced CT/MRI (CECT/CEMRI)] of the base of skull to thoracic inlet, chest X-ray PA view or high-resolution CT (HRCT) in case of doubt; and pre-radiation therapy dental evaluation.

**Figure 1:**
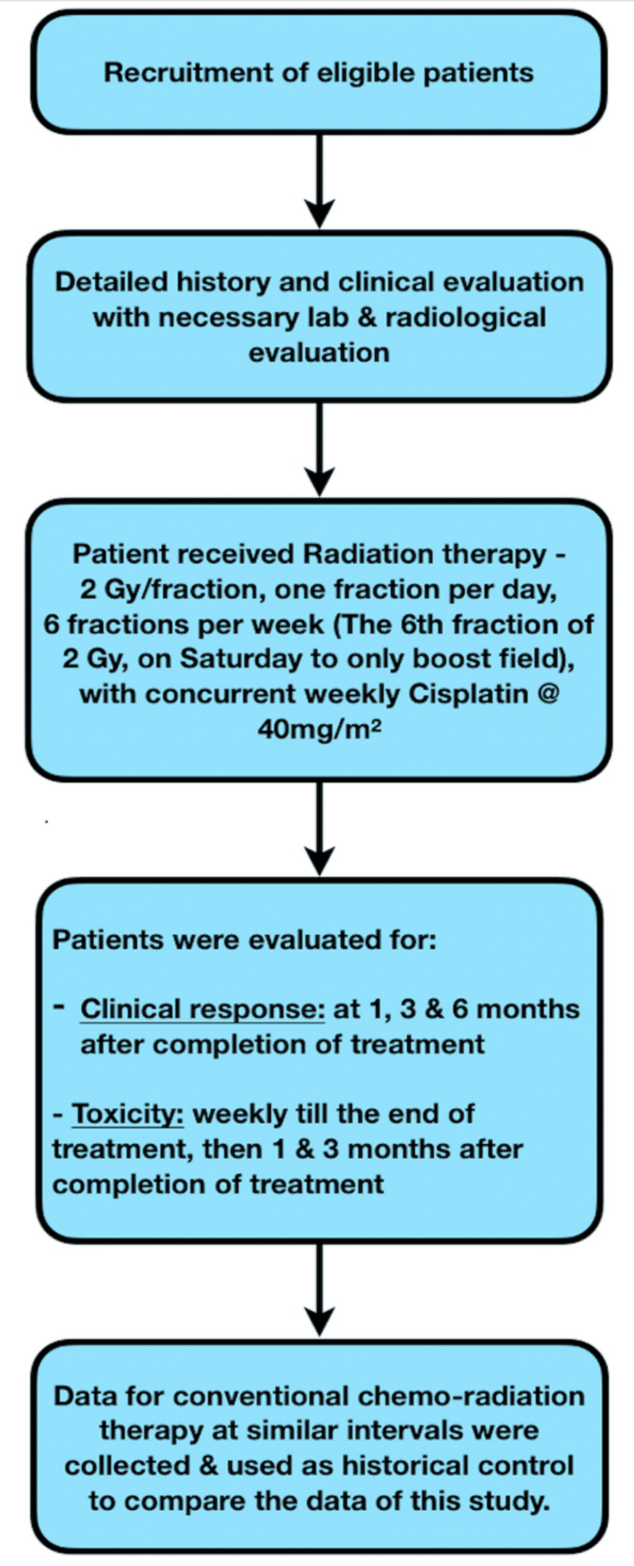
Methodology flowchart

The eligible patients were scheduled to receive 2 Gy per fraction, one fraction per day to a total dose of 66-70 Gy in 33-35 fractions six days a week over 5.5 weeks. Patients were planned with bilateral parallel opposed face and neck fields in three phases with Cobalt-60 teletherapy machine - Bhabhatron-II, by the source-to-surface distance of 80 cm. The first phase included the gross disease, involved nodes, and the lymph nodal stations with a higher propensity of microscopic spread to a dose of 44 Gy in 22 fractions. Simultaneously, the patient was planned for the third phase i.e., the boost phase which included the gross disease and involved nodes with a 2 cm margin, sparing the spinal cord. The radiation dose to the boost phase was 10 Gy in five fractions and was delivered only on Saturdays along with the first phase delivered from Monday to Friday. After the completion of the first phase, i.e., 44 Gy in 22 fractions, cord sparing was done by replanning for the second phase, to a total dose of 66-70 Gy at 2 Gy per fraction delivered from Monday to Friday depending on the patient's tolerability. The patients received concurrent chemotherapy with cisplatin at 40 mg/m^2^ weekly. The flowchart of the treatment scheme is presented in Figure 1.

Follow-up

Response evaluation was done at one, three, and six months post-treatment completion as per Response Evaluation Criteria in Solid Tumors 1.128 criteria (RECIST-1.128) by CECT/CEMRI base of the skull to the thoracic inlet at the third/sixth month. For toxicity evaluation during the treatment, patients were reviewed every week till completion of treatment, and toxicity was documented every week, as per Radiation Therapy Oncology Group (RTOG) criteria and it was managed accordingly. Evaluation for toxicity continued after completion of the treatment at one and three months.

Statistical analysis

The collected data were entered into Microsoft Excel and then analyzed and statistically evaluated using the SPSS Statistics version 25 (IBM Corp., Armonk, NY). The normality of each variable was assessed by using the Kolmogorov- Smirnov test and the Shapiro-Wilk test. Quantitative data were expressed in mean and standard deviation (SD), or median with interquartile range (IQR) and depended on normal distribution. Qualitative data were expressed in percentages.

## Results

Patient characteristics

The baseline characteristics of the enrolled patients are summarized in Table 1.

**Table 1:** Baseline characteristics SD: standard deviation

Baseline characteristics	Values	
Age group, years, n (%)		
30-40	7 (15.56%)	
41-50	13 (28.89%)	
51-60	15 (33.33%)	
61-70	10 (22.22%)	
Mean age ± SD, years	50.65 ± 10.4	
Median age (25th-75th percentile), years	51 (42-59)	
Range, years	30-70	
Gender, n (%)		
Female	1 (2.22%)	
Male	44 (97.78%)	
Site, n (%)		
Hypopharynx	1 (2.22%)	
Larynx	7 (15.56%)	
Supra glottis	1 (2.22%)	
Glottis	6 (13.33%)	
Oral cavity	24 (53.33%)	
Alveolus	2 (4.44%)	
Buccal mucosa	3 (6.67%)	
Floor of mouth	1 (2.22%)	
Gingivo buccal sulcus	1 (2.22%)	
Hard palate	1 (2.22%)	
Tongue	16 (35.55%)	
Oropharynx	13 (28.89%)	
Base of tongue	8 (17.78%)	
Soft palate	2 (4.44%)	
Tonsil	3 (6.67%)	
T stage, n (%)		
T1	1 (2.22%)	
T2	1 (2.22%)	
T3	17 (37.78%)	
T4a	25 (55.56%)	
T4b	1 (2.22%)	
N stage, n (%)		
N0	2 (4.44%)	
N1	10 (22.22%)	
N2a	2 (4.44%)	
N2b	11 (24.44%)	
N2c	20 (44.44%)	
Stage, n (%)		
III	7 (15.56%)	
IV	38 (84.44%)	

Outcome

In this study, out of the 51 patients, five patients defaulted after starting the treatment. Hence, they were excluded from the study. One patient died during the treatment. The remaining 45 patients were able to complete their treatment as per protocol, within the time frame. The median follow-up in the study was six months.

**Figure 2:**
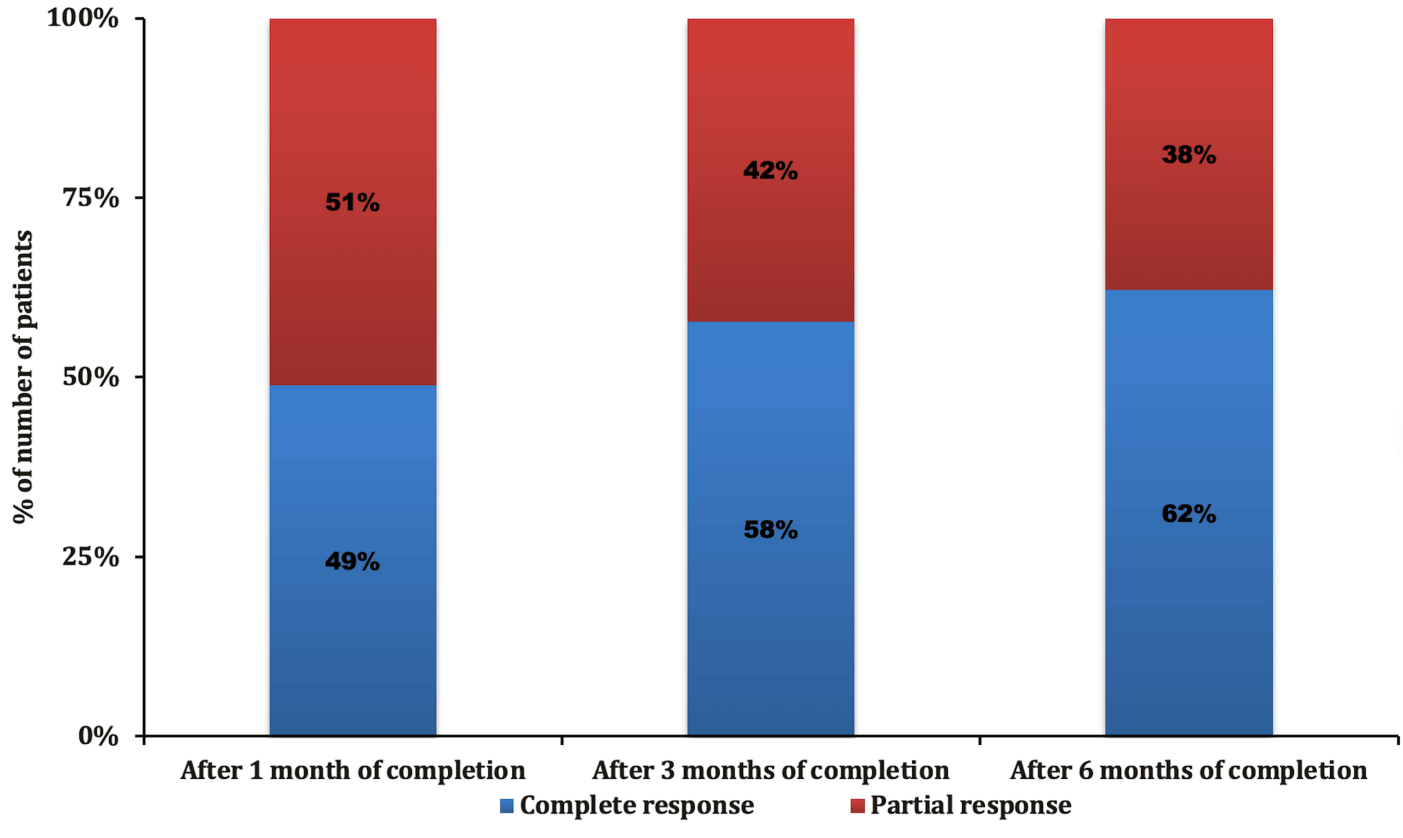
Response distribution

After the first month of treatment completion, 22 patients (48.89%) had a complete response and 23 patients (51.11%) had a partial response. After the third month of treatment completion, 26 patients (57.78%) had a complete response and 19 patients (42.22%) had a partial response. Finally, after the sixth month of treatment completion, 28 patients (62.22%) had a complete response, 17 patients (37.78%) had partial response/residual disease, and there was no patient with stable disease or progressive disease at the six-month follow-up. The response at the first, third, and sixth month post-treatment completion is illustrated in Figure 2.

In patients with residual tumors, salvage surgery or palliative treatment was offered, depending on the performance status and symptoms, and after a multidisciplinary tumor board discussion. The median duration of treatment was 50 days (42-69 days). This study had the benefit of a reduced overall treatment time of eight days with a median treatment time of 50 days (42-69 days) in contrast with conventional chemoradiation, where the overall treatment time is generally 58 days (50-69 days) [13-14].

**Figure 3:**
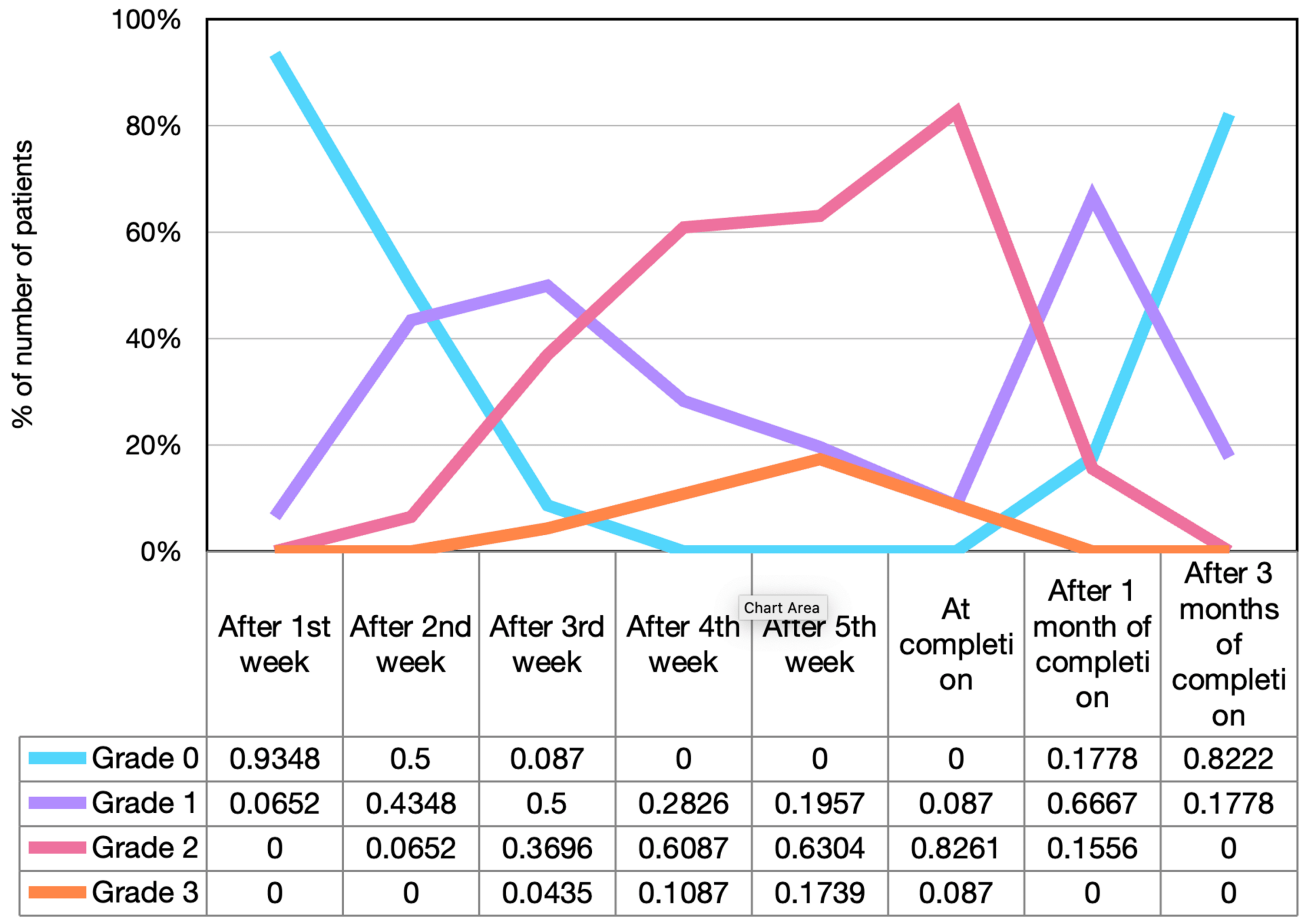
Mucositis toxicity distribution

**Figure 4:**
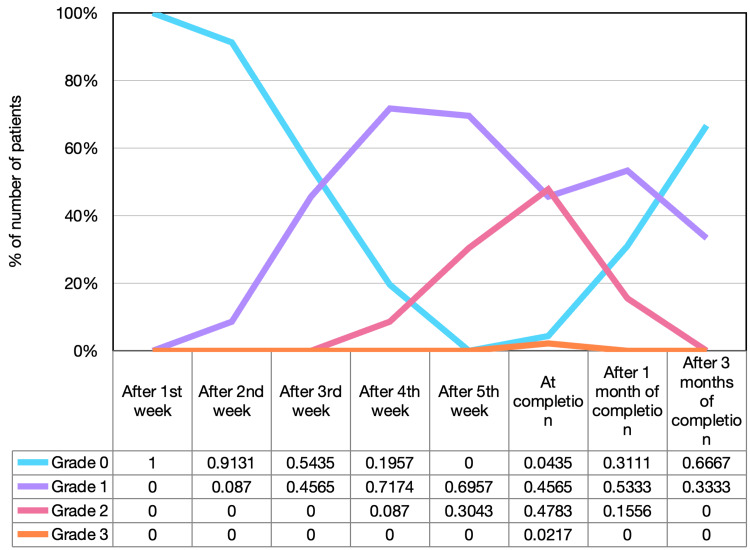
Dermatitis toxicity distribution

The toxicities developed during the treatment and, the first and third-month post-treatment completion are demonstrated in Figures 3-4 according to RTOG criteria. After the completion of accelerated concomitant chemoradiation, four patients (8.89%) had grade 1 mucositis, 37 patients (82.22%) had grade 2 mucositis, and four patients (8.89%) had grade 3 mucositis. On the other hand, only one patient (2.22%) developed grade 3 dermatitis at completion; 21 patients (46.67%) had grade 2 dermatitis, 21 patients (46.67%) had grade 1 dermatitis, and two patients (4.44%) had grade 0 dermatitis.

After three months of treatment completion, all cases of grade 2 and 3 mucositis were healed and only eight patients (17.78%) had grade 1 mucositis whereas mucositis was resolved in the remaining 37 patients (82.22%). Of note, 15 patients (33.33%) still had grade 2 dermatitis and the remaining 30 patients (66.67%) had grade 1 dermatitis. Toxicity distribution during the treatment is illustrated in Figures 3-4.

## Discussion

In this study, 51 patients of unresectable locally advanced HNSCC were recruited to receive accelerated concomitant chemoradiation, out of which 45 completed the treatment and full follow-up. Of note, 44 patients (97.78%) were male and only one patient (2.22%) was female. This trend could be due to the higher prevalence of tobacco use among males than females in India [16]. The most common site was the oral cavity (n=24, 53.33%). Most of the patients had stage IV cancer, which can be attributed to the lack of awareness and negligence among the relatively illiterate patient population attending the government healthcare facility, due to which most of the patients present very late with advanced disease [17].

Concomitant boost radiation therapy was considered for the study because of the radiobiological aspects of accelerated radiation fractionation, which include (1) reduction in tumor cell repopulation by reducing the overall treatment time; (2) enhanced tumor cell kill by reducing the time for repair between sessions; (3) increased intensity of treatment with accelerated fractionation leads to higher biologically effective dose (BED) to tumor cells without a proportionate increase in damage to normal tissue; and (4) accelerated fractionation reduces the time for tumor reoxygenation, making cancer cells more susceptible to damage by CTRT [4-12]. Several meta-analyses such as DAHANCA 6 and 7 [15], RTOG-9003 [18], and MARCH meta-analysis [19] have compared conventional fractionation with accelerated radiation fractionation, reporting improved progression-free survival (PFS) and five-year locoregional control with accelerated radiation with increased incidence of acute toxicities but no increase in late toxicities.

We used a similar protocol as DAHANCA 6 and 7 in our study. DAHANCA 6 and 7 compared the conventional radiation schedule (five fractions per week) to accelerated radiation (six fractions per week) with the same total dose and fraction (66-68 Gy in 33-34 fractions) of radiation in both the groups of HNSCC. With the benefit of an eight-day faster completion of accelerated radiation schedule, there was a statistically significant benefit in five-year locoregional control rates (75% vs. 66% five-year actuarial rate, p=0.0006) in the accelerated radiation group [15]. Another study (IAEA-ACC study) by the same researchers (Overgaard et al.) also compared five versus six fractions of radiotherapy per week for HNSCC. It was a randomized multi-centric trial involving multiple developing countries with the same protocol as DAHANCA 6 and 7. It concluded that with the benefit of a seven-day decrease in overall treatment time, accelerated fractionation of radiation therapy resulted in significantly better locoregional control at five years (42% vs. 30%) [12].

In our study, 28 patients (62.22%) had a complete response and 17 patients (37.78%) had a partial response after six months of treatment completion. In a similar study by Gupta et al., out of 30 patients who received six fractions of radiation per week, 14 patients (46.7%) had a complete response and 10 patients (33.3%) had a partial response at six weeks post-treatment completion [13]. In another similar study by Baral et al. with a similar treatment protocol and follow-up, out of 30 patients receiving six fractions of radiation per week, 75% had a complete response [14]. The variation in the responses in different studies can be attributed to the difference in the proportion of stage III and IV patients recruited. Stage IV patients receiving six fractions per week accounted for only 25% of DAHANCA 6 and 7, 36% in the IAEA-ACC study, and 56.7% in the study by Gupta et al. Whereas, in our study, 84.44% of patients were stage IV.

In all the studies comparing six fractions per week with five fractions per week, toxicities were also documented; the increase in toxicities was expected since treatment time decreased with six fractions of radiation per week. DAHANCA 6 and 7 reported 53% vs. 33% of confluent mucositis in six vs. five fractions of radiation per week, respectively, and it persisted for a longer duration in the group of six fractions per week but all healed within three months of starting the treatment. Late radiation-related complications did not differ significantly between both groups. IAEA-ACC study also reached the same conclusion about confluent mucositis with a frequency of 10% and 5% of confluent mucositis in six and five fractions of radiation per week respectively, and they also reported 20% vs. 11% of severe skin reactions in six vs. five fractions of radiation per week.

In our study, the maximum toxicity occurred at the completion of treatment, after which it started resolving on monthly follow-ups. At the completion of treatment, four patients (8.70%) had grade 1, 38 patients (82.61%) had grade 2, and four patients (8.70%) had grade 3 oral mucositis. Two patients (4.35%) had grade 1 dermatitis, 21 patients (45.65%) had grade 2 dermatitis, 22 patients (47.83%) had grade 3 dermatitis, and only one patient (2.17%) had grade 4 dermatitis at the completion of treatment. After three months of treatment completion, all the grades of dermatitis resolved except for grade 1 dermatitis, which was still present in 15 patients (33.33%). In a similar study by Gupta et al., 33.3% had grade 2 oral mucositis, 33.3% had grade 3 oral mucositis, and 10% had grade 4 oral mucositis out of the 30 patients who received six fractions of radiation per week. In another similar study by Baral et al., 57.1% of patients in the six fractions per week group developed grade 3 and 4 oral mucositis, and 28.6% developed grade 3 and 4 dermatitis.

Data on response and toxicity were obtained for conventional chemoradiation of five fractions of radiation per week, at similar intervals from these similar studies by Gupta et al. and Baral et al. [13,14]. Then, a comparison was done between the data of response and toxicities of six fractions of radiation per week (concomitant boost group) vs. five fractions of radiation per week (conventional chemoradiation); 28 patients (62.22%) in the concomitant boost group and 18 patients (60%) in the conventional chemoradiation group had complete responses, which was statistically not significant (p=0.846). Seventeen patients (37.78%) in the concomitant boost group had partial response compared to eight patients (26.67%) in the conventional chemoradiation group, which was also statistically not significant (p=0.317). A comparison of toxicity was also done between the two groups, and the difference was statistically significant for acute toxicity (mucositis) (p=0.0003). This signifies that there was a statistically significant greater toxicity in patients receiving six fractions of radiation per week as compared to the ones receiving only five fractions of radiation per week, which were manageable.

This study has a few limitations. It was not a randomized study, and it had a small sample size and a shorter duration of follow-up. However, these limitations should not overshadow the advantages of this study: shorter duration of the treatment without the requirement of any additional resources; and accommodating a greater number of patients for treatment leading to efficient utilization of resources with similar response rates and manageable acute toxicities, especially in low and middle-income countries, with a huge burden of cancer patients due to the prevalent use of chewable tobacco, bidi, cigarette, pan masala, etc., without adequate cancer treatment facilities.

## Conclusions

Accelerated concomitant chemoradiation had a 62.22% complete remission rate with manageable acute mucositis and dermatitis, which resolved in 82% and 67% respectively within three months. The overall treatment time in patients of accelerated concomitant chemoradiation was substantially lower (an advantage of eight days); hence, accelerated concomitant chemoradiation can be the new standard of care for locally advanced HNSCC. However, there is scarce data in the literature regarding accelerated concomitant chemoradiation regimens. Studies with larger sample sizes and longer follow-ups are needed to validate our findings and gain deeper insights into the feasibility of accelerated concomitant chemoradiation.

## References

[1] Sarnath D, Khanna A (2022). Current status of cancer burden: global and Indian scenario. Biomed Res J.

[2] Saunders MI (1999). Head and neck cancer: altered fractionation schedules. Oncologist.

[3] Krstevska V (2009). Radiotherapy and chemotherapy in locally advanced head and neck squamous cell carcinoma. J BUON.

[4] Withers HR, Taylor JM, Maciejewski B (1988). The hazard of accelerated tumor clonogen repopulation during radiotherapy. Acta Oncol.

[5] Bentzen SM, Thames HD (1991). Clinical evidence for tumor clonogen regeneration: interpretations of the data. Radiother Oncol.

[6] Dubben HH (1994). Local control, TCD50 and dose-time prescription habits in radiotherapy of head and neck tumours. Radiother Oncol.

[7] Thames HD, Bentzen SM (1995). Time factor for tonsillar carcinoma. Int J Radiat Oncol Biol Phys.

[8] Fowler JF, Lindstrom MJ (1992). Loss of local control with prolongation in radiotherapy. Int J Radiat Oncol Biol Phys.

[9] Pinto LH, Canary PC, Araújo CM, Bacelar SC, Souhami L (1991). Prospective randomized trial comparing hyperfractionated versus conventional radiotherapy in stages III and IV oropharyngeal carcinoma. Int J Radiat Oncol Biol Phys.

[10] Horiot JC, Le Fur R, N'Guyen T (1992). Hyperfractionation versus conventional fractionation in oropharyngeal carcinoma: final analysis of a randomized trial of the EORTC cooperative group of radiotherapy. Radiother Oncol.

[11] Fu KK, Pajak TF, Trotti A (2000). A Radiation Therapy Oncology Group (RTOG) phase III randomized study to compare hyperfractionation and two variants of accelerated fractionation to standard fractionation radiotherapy for head and neck squamous cell carcinomas: first report of RTOG 9003. Int J Radiat Oncol Biol Phys.

[12] Overgaard J, Mohanti BK, Begum N (2010). Five versus six fractions of radiotherapy per week for squamous-cell carcinoma of the head and neck (IAEA-ACC study): a randomised, multicentre trial. Lancet Oncol.

[13] Gupta Y, Garg P, Banipal RPS, Bedi N (2017). Concomitant boost on Saturdays in radiotherapy for locally advanced oral cavity and oropharyngeal cancers. Int J Contemp Med Res.

[14] Baral S, Adhikari A, Pandit S, Silwal S, Acharya B, Shah A, Jha AK (2014). Five versus 6 fractions per week of radiation therapy in squamous cell carcinoma of head and neck. Int J Radiat Oncol Biol Phys.

[15] Overgaard J, Hansen HS, Specht L (2003). Five compared with six fractions per week of conventional radiotherapy of squamous-cell carcinoma of head and neck: DAHANCA 6 and 7 randomised controlled trial. Lancet.

[16] Pahari S, Barman D, Talukdar R (2023). Tobacco usage in India: a meta-analysis of evidence drawn from regional studies between 2010 and 2022. Trop Med Int Health.

[17] Sahu DP, Subba SH, Giri PP (2020). Cancer awareness and attitude towards cancer screening in India: a narrative review. J Family Med Prim Care.

[18] Beitler JJ, Zhang Q, Fu KK (2014). Final results of local-regional control and late toxicity of RTOG 9003: a randomized trial of altered fractionation radiation for locally advanced head and neck cancer. Int J Radiat Oncol Biol Phys.

[19] Lacas B, Bourhis J, Overgaard J (2017). Role of radiotherapy fractionation in head and neck cancers (MARCH): an updated meta-analysis. Lancet Oncol.

